# High‐rate ethanol production at low pH using the anaerobic granular sludge process

**DOI:** 10.1002/bit.27708

**Published:** 2021-03-03

**Authors:** Jelmer Tamis, Bart Joosse, Kasper de Leeuw, Robbert Kleerebezem

**Affiliations:** ^1^ Department of Emerging Technologies Paques BV Balk The Netherlands; ^2^ Department of Biotechnology Delft University of Technology Delft The Netherlands; ^3^ Present address: Waterschap Brabantse Delta Breda The Netherlands; ^4^ Present address: Department of Agrotechnology and Food Sciences Wageningen University and Research Wageningen The Netherlands

**Keywords:** ethanol, granular sludge, open/mixed culture fermentation, resource recovery, VFA

## Abstract

In this study, we investigated the operational performance and product spectrum of glucose‐fermenting anaerobic granular sludge reactor at pH 4. A selective environment for the growth of granules was implemented by the introduction of a 2 min settling phase, a hydraulic retention time of 6 h and a solid retention time of 12 ± 3 days. The fermentation products were ethanol, lactate, and volatile fatty acids (VFA) with yields of 0.55 ± 0.03, 0.15 ± 0.02, and 0.20 ± 0.04 gram chemical oxygen demand (gCOD)/gCOD glucose, respectively. The obtained product spectrum was remarkably different from the VFA‐dominated product spectrum reported in a previous study when the same system was operated at higher pH (4.5–5.5). The shift in product spectrum coincided with a shift in the microbial community structure with the dominance of eukaryotic *Candida tropicalis, Pichia jaroonii*, and prokaryotic *Lactobacillus* species instead of the *Clostridia* species obtained at higher pH‐values. The control of the microbiomes and the associated product spectra provides bioprocess engineers with the option to tailor a suitable precursor compound mixture for subsequent chain elongation fermentation or PHA biopolymer production.

## Graphical abstract

Comparison between the product spectra on COD basis of the effluent of the bioreactor operated at pH 4.5‐5.5 in an earlier study (Tamis et al., 2015) and pH 3.5‐4.0 in this study.

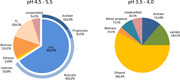

## 1 INTRODUCTION

Anaerobic fermentation is a method to convert the multitude of compounds present in organic residue streams into a more defined range of products while largely maintaining the energy content of the substrates. The end‐product in anaerobic fermentations of organic matter as governed by thermodynamics is methane‐containing biogas (Kleerebezem et al., 2015). However, it is possible to direct the fermentations toward potentially more valuable intermediate products such as carboxylates and alcohols. Interestingly, it is challenging to control the product spectrum of waste stream fermentations because the complexity and unsterile nature of these substrates prevent direct control over the microorganisms that grow in these processes. Instead, process conditions determine which species have a competitive growth advantage (Kleerebezem & van Loosdrecht, 2007). Although volatile fatty acids (VFA) and ethanol are usually the main products in these *open* anaerobic fermentations (sometimes referred to as mixed culture fermentations), current understanding of how environmental factors, such as pH, temperature, solid retention time (SRT), and so on, influence the product spectrum of open cultures is limited.

In this study, we investigate the effect of low pH on the product spectrum of anaerobic glucose fermentation in a SBR. Anaerobic fermentation at low pH is challenging from a microbe's perspective due to the toxicity of the carboxylic acids produced. The diffusion of acids through the cell membrane at a higher rate than their conversion by the cell necessitates constant export by the microorganisms, causing an energy‐draining cycle (Mira et al., 2010). Under these circumstances, growth rates may be relatively low, and biomass retention may be used to retain the slow‐growing biomass. In a previous study (Tamis et al., 2015) efficient biomass retention and VFA production was achieved in a granular sludge type fermentation at pH 4.5–5.5. Since the variation in product spectrum between pH 4.5 and 5.5 was found to be rather small, we decided to evaluate whether this trend continues or is broken at lower pH. In an earlier publication, it was proposed that at lower pH, microorganisms cannot sustain themselves if they produce VFA and therefore should switch to the production of compounds that do not lead to exhibit increased toxicity with lower pH, that is, ethanol (Rodríguez et al., 2006). Contrary to these predictions, studies on open culture fermentation of sugar at moderately low pH (4.5–5.5) have reported acetate and butyrate and main products instead of ethanol (Fang & Liu, 2002; Temudo et al., 2007). Other studies have attempted high hydrogen partial pressure as a potential driver for ethanol production but found consumption of ethanol in favor of chain elongation of fatty acids (Steinbusch et al., 2010; Wang et al., 2018). Industrial yeast fermentations seem to rely on the addition of dedicated pure cultures to establish high ethanol yields (Siqueira et al., 2008). In this manuscript, we investigate the ethanol production in open cultures using low pH as a selective pressure.

## 2 METHODS

**Figure 1:**
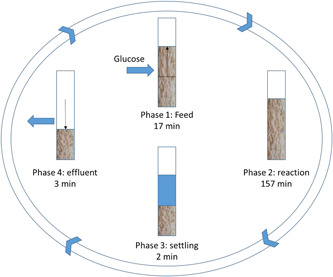
Overview of the operational cycle of the bioreactor used for the anaerobic fermentation of glucose [Color figure can be viewed at wileyonlinelibrary.com]

The same reactor system as reported in a previous study (Tamis et al., 2015) was operated, but the operational pH was decreased to 4.0 and 3.5. The reactor setup and operational parameters were largely identical to the earlier reported system: briefly, the reactor (*V_L_* = 2.6 L) was operated as a sequencing batch reactor (SBR) at 30°C. The reactor was inoculated with an anaerobic culture obtained from the previous experiment running at pH 4.5. The system had an operational cycle of 3 h in total, consisting of four phases as follows (Figure 1). Phase 1 was a feed phase of 17 min (1.3 L/cycle containing 10.5 g/L glucose and nutrients (g/L) NH_4_Cl 0.678; KH_2_PO_4_ 0.127; MgSO_4_·7H_2_O 0.059; KCl 0.020; ethylenediaminetetraacetic acid 0.036; ZnSO_4_·7H_2_O 0.012; CoCL_2_·6H_2_O 0.0009; MnCl_2_·4H_2_O 0.0029; CuSO_4_·5H_2_O 0.0009; FeSO_4_·7H_2_O 0.0028; (NH_4_)_6_Mo_7_O_24_·4H_2_O 0.0006; CaCl_2_·H_2_O 0.0041). Phase 2 was a reaction phase of 157 min; phase 3 was a settling phase of 2 min, and phase 4 was a 3 min effluent phase in which half of the reactor liquid (1.3 L/cycle) was decanted, resulting in a hydraulic retention time (HRT) of 6 h and a chemical oxygen demand (COD) loading rate of 45 gram chemical oxygen demand (gCOD)/(L·d).

Samples were taken from the liquid phase and analyzed by high performance liquid chromatography (HPLC) and gas chromatography for ethanol and VFA quantification using the protocols described in an earlier publication (Tamis et al., 2015). The feed glucose concentration was established by weighing the amount of glucose with respect to the total feed volume prepared. The solid concentrations were monitored to obtain biomass yields, sludge volume index (SVI), and SRT. Kinetic and stoichiometric parameters were obtained by calculation from steady‐state data and by calibration of a mathematical model that was published earlier (Tamis et al., 2015) using the data from a detailed cycle measurement. The microbial community structure was evaluated using denaturing gradient gel electrophoresis (DGGE) using eukaryotic and prokaryotic primers (Muyzer, 1999). For the plating experiments, 1% agar plates with reactor growth medium were incubated in an anaerobic jar that was flushed with nitrogen gas to ensure anaerobic conditions and placed at 30°C for 24 h. Plates were primed with the substrate and the pH was set to 3.5–4 (mimicking reactor conditions). Plates were inoculated and incubated in an anaerobic jar that was placed in a shaker at 30°C for 7 days. Colony growth was visually checked every day. More detailed information about the plating method is provided in Appendix 2.

## 3 RESULTS

### 3.1 Start‐up and granulation

A sequencing batch granular sludge bioreactor was inoculated with granular sludge grown at pH 4.5. The operational pH in this process was lowered to 4.0. The first response of the system was degeneration of the granular structure of the sludge and the settling time and cycle length needed to be set at 30 min and 24 h initially. In subsequent weeks, the settling time and cycle length could be gradually decreased and fast settling biomass established in the system while removing slow‐settling biomass via the effluent of the system. During start‐up, the length of the reaction phase was manually adjusted to assure complete conversion of glucose before initiation of the settling and effluent phases. The complete glucose conversion was validated by HPLC measurement of the effluent.

**Figure 2:**
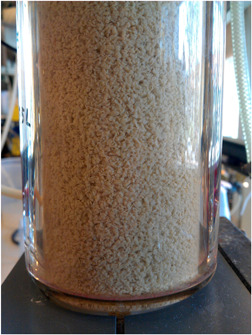
Impression of the 0.5–2 mm anaerobic granules in the reactor [Color figure can be viewed at wileyonlinelibrary.com]

**Figure 3:**
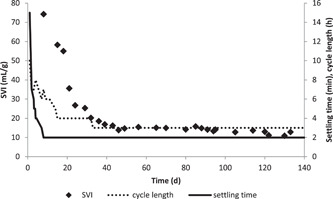
The sludge volume index of the biomass inside the reactor as well as settling time and cycle lengths. The settling time and later the cycle length were decreased during start‐up. The sludge became gradually more compact showing a decreasing SVI. SVI, sludge volume index

This start‐up procedure was completed on day 33; from this day until the end of the experiment, the reactor was operated with a settling time of 2 min and a cycle length of 3 h. Granule formation became visible around day 21. The granules were ovoid‐shaped with sizes varying between 0.5 and 2 mm (Figure 2). The SVI declined rapidly from more than 75 to 17 ml/gVSS during start‐up and became relatively stable after the start‐up period with a decline in SVI from 17 to 12 ml/gVSS (Figure 3).

### 3.2 Steady state

The reactor performance was monitored over a period of 136 days (1000 cycles of 3 h) at pH 4.0. After the start‐up period of 33 days, a steady state was achieved judged by the relatively constant biomass‐specific conversion rates, SVI, SRT, and the product spectra (Table 1). The average concentration of VSS in the bioreactor during the steady‐state period was 14 ± 2 g/L or 35 ± 5 gVSS in 2.6 L liquid. The concentrations of total suspended solids and volatile suspended solids (VSS) in the effluent were 0.31 ± 0.15 g/L and 0.29 ± 0.15 g/L, respectively. Herewith the net removal of solids was 3.0 ± 0.9 gVSS/d (2.3 ± 0.9 gVSS/d via the effluent and 0.7 gVSS/d via manual solids removal and sampling), resulting in an SRT of 12 ± 3 days.

**Table 1:** Comparison of the characteristics of the system during steady‐state (average ± *SD* over data set) for earlier published operation at pH 4.5 and higher (pH 4.5 is taken as a representative state) and operation at pH 4.0 and low (this study)

pH	4.5	4.0	3.5	
	Tamis et al. (2015)	This study	This study	
SVI	22 ± 3	14 ± 2	17 ± 3	mL/gVSS
Y_X_	0.13 ± 0.01	0.03 ± 0.01	0.04 ± 0.01	gCOD/gCOD
Y_ethanol_	0.02 ± 0.01	0.55 ± 0.03	0.61 ± 0.04	gCOD/gCOD
Y_VFA_	0.66 ± 0.02	0.20 ± 0.04	0.13 ± 0.01	gCOD/gCOD
Y_lactate_	0.00 ± 0.00	0.15 ± 0.02	0.13 ± 0.02	gCOD/gCOD
q_s_^max^	0.7	0.24 ± 0.03	0.29 ± 0.04	gCOD/(gVSS·h)
SRT	2 ± 1	12 ± 3	10 ± 3	d

Abbreviations: gCOD, gram chemical oxygen demand; SRT, solid retention time; SVI, sludge volume index; VFA, volatile fatty acid, VSS, volatile suspended solids.

In the next period, the reactor pH was lowered to 3.5 and the new steady state (90 days) was compared to the one at pH 4.0. No major differences between both states were observed (Table 1). Since these values were largely comparable between pH 4 and pH 3.5, only a detailed description of the system at pH 4.0 is provided in this paper.

### 3.3 Product spectrum

**Figure 4:**
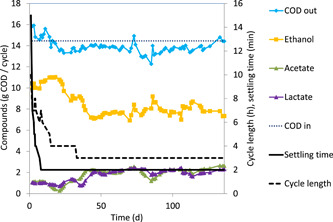
Profile of the main products in the effluent of the anaerobic fermentation for the duration of the whole experiment (136 days). The influent COD was determined by weighing the amount of glucose and was constant. The sum of the identified product (COD out) was close to the influent COD (94% ± 4%). COD, chemical oxygen demand [Color figure can be viewed at wileyonlinelibrary.com]

The main products in the effluent were ethanol, lactate, and acetate (Figure 4). Evaluation of the COD and carbon balances showed that the total product constituted 94% ± 4% of the influent for both COD and carbon. Analysis of the off‐gas showed that hydrogen and methane production was negligible (responsible for less than 1% of the total COD conversion). The base dosage that was needed to keep a constant pH of 4.0 was 0.27 ± 0.03 mol NaOH/(mol glucose). This was within 10% of the amount estimated from the charge balance (based on the production of dissociated organic acids and consumption of ammonium (Tamis et al., 2015). Proton release could be attributed to the produced dissociated organic acids for 88% and to the uptake of ammonium for 12%.

### 3.4 Kinetic and stoichiometric characterization

**Figure 5:**
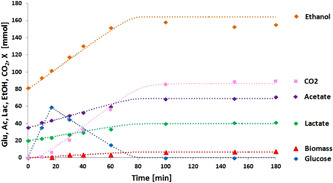
Product formation during a representative cycle (day 121) fitted with a data evaluation model in which an assumed lumped stoichiometry for glucose conversion was used. The symbols represent measured data points, while the dotted lines represent the model [Color figure can be viewed at wileyonlinelibrary.com]

After reaching steady state, the conversions during a representative cycle at pH 4.0 were investigated (day 121 of operation). It was observed that product formation occurred only during the first part (80 min) of the cycle when glucose was still present in the reactor (Figure 5). The overall volumetric glucose conversion rate of the system was 45 gCOD/(L d), but this included the inactive period of the cycle during which glucose was depleted. Consequently, the actual rates of glucose conversion were significantly higher up to a value of 100 gCOD/(L d).

The data from the cycle experiments were used as a basis for a process model (Supporting Information Material), and a good representation of the experimental data could be obtained, indicating that one lumped reaction sufficed to describe the actual conversions observed. The stoichiometry and kinetic parameters acquired from the model were comparable to the long‐term operation steady‐state averages (Appendix 1).

### 3.5 Microbial community structure

**Figure 6:**
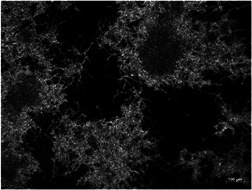
Microscope image of crushed granular biomass observed on day 23 with ×100 magnification. Separating the cells via crushing proved difficult due to the hyphae entanglement

**Figure 7:**
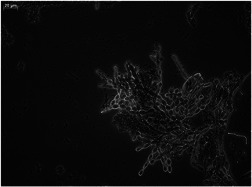
Microscope image of a small fragment of a crushed granule observed on day 87 with ×400 magnification. The image shows elongating and branching yeast hyphae with aggregates of bacteria in between

Microscopic and DGGE analysis revealed the presence of both prokaryotic and eukaryotic species. During the whole operation period, *Lactobacillus* sp. were detected, which are known for anaerobic fermentation of glucose into lactate and ethanol (De Vos et al., 2005). Moreover, some *Lactobacillus* species are also able to produce acetate during fermentation with less reduced substrates (Miyamoto et al., 2005). Furthermore, *Propionibacterium* sp. were detected throughout the operation. A temporary presence of *a Pectinatus* sp. was detected concomitant with a propionate production peak during start‐up. The eukaryotes in the reactor were identified as species from the *Candida*/*Pichia* genus, generally known for ethanol production (Parekh et al., 1986). Microscopic observations showed that the granules consisted of a large fraction of hyphae growing yeasts and another fraction of smaller bacteria (Figures 6 and 7).

**Figure 8:**
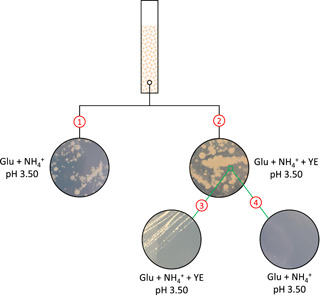
Schematic overview of the plate experiments with granular biomass: (1) on minimal medium only yeast could grow; (2) both bacteria and yeast grew when yeast extract was added; (3) the transfer of bacterial colonies from “2” to plates with yeast extract resulted in growth, while (4) transfer of bacterial colonies from “2” to the minimal medium resulted in no growth [Color figure can be viewed at wileyonlinelibrary.com]

In an attempt to clarify the coexistence of the eukaryotic and bacterial species, several plating experiments were carried out. It was observed that the bacteria could not grow on plates that were composed of influent growth medium and 1% agar, but yeast could (Figure 8, route 1). To check whether bacteria could only grow in coexistence with yeast or a compound produced by yeast, reactor biomass was transferred to plates composed of growth medium and 5 g/L yeast extract (Figure 8, route 2). On these plates, both yeast and bacterial colonies were observed. When bacterial colonies were subsequently transferred to growth medium plates without yeast extract (Figure 8, route 4) no bacterial colonies appeared, while bacterial colonies did appear when plates supplemented with the yeast extract were used (Figure 8, route 3).

## 4 DISCUSSION

### 4.1 Anaerobic granular sludge at low pH

Granular sludge systems offer the possibility to operate at relatively adverse conditions while maintaining biological activity where CSTR systems fail. For example, effective operation at pH 4 was hardly possible in a CSTR (Temudo et al., 2008). Furthermore, the use of granular sludge systems for the production of VFA and ethanol offers several advantages over CSTR systems from a practical point of view: dissolved products and biomass solids are separated in the process, and high volumetric conversion rates can be achieved even at low substrate concentrations. An advantage of operation at a low pH value is the reduction of the amount of base chemicals required to maintain the desired pH. For example, the theoretical amount of NaOH (per mol of VFA produced) that would be required to maintain pH in the reactor at pH 4 is approximately 0.16 mol NaOH while it is 0.66 mol NaOH at pH 5. Further studies should explore the limits of granular sludge type of systems, for example, anaerobic fermentation at pH 3 and below. Moreover, operating at higher VFA concentrations would be interesting to investigate.

### 4.2 Product spectrum and microbial community structure

**Figure 9:**
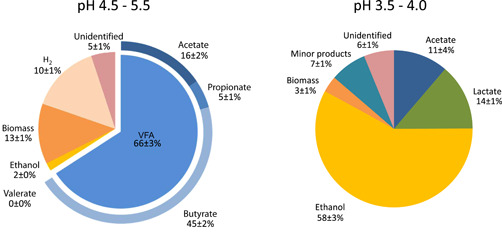
Comparison between the product spectra on chemical oxygen demand basis of the effluent of the bioreactor operated at pH 4.5–5.5 in an earlier study (Tamis et al., 2015) and pH 3.5–4.0 in this study. VFA, volatile fatty acid [Color figure can be viewed at wileyonlinelibrary.com]

**Figure 10:**
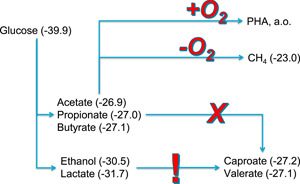
Conversion pathways for the anaerobic fermentation of glucose and the subsequent options for industrial valorization; between brackets, the Gibbs free energy per electron (kJ/mol) calculated from the reaction of each compound to CO_2_ and H_2_O. The resulting Gibbs energies show that both ethanol and lactate offer the opportunity for further conversion to medium chain length fatty acid, while volatile fatty acid can only be converted either to methane (anaerobically) or to molecules that are produced aerobically, like PHA [Color figure can be viewed at wileyonlinelibrary.com]

With ethanol as the main product, the product spectrum in the reactor operated at pH 3.5–4.0 was significantly different (Figure 9) from the VFA‐dominated spectrum obtained previously in the same reactor at pH 4.5–5.5 (Tamis et al., 2015) (Figure 10).

While no significant change was observed in the product spectrum when lowering the pH from 5.5 to 4.5, a further decrease in the pH to 4.0–3.5 resulted in a dramatic shift. This coincided with the emergence of eukaryotic yeast species, known for the production of ethanol and tolerance to higher concentrations of organic acids and ethanol. The observed switch to ethanol is likely related to natural environments where there is ample fermentable sugars and only a limiting amount of alkalinity, for example, rotten fruits, a niche where yeasts have specialized in evolving a tolerance possibly by adaptations in the eukaryotic cell membrane making it less permeable to these compounds (Dashko et al., 2014; Gostinčar et al., 2009; Mira et al., 2010). The eukaryotes were accompanied by a bacterial side population of *Lactobacilli*. The stable production of lactate throughout the operation indicated a second major difference between operation at low pH (3.5–4) (this study) and higher pH (4.5–5.5) (Tamis et al., 2015) where transient amounts of lactate were converted to propionate and acetate. The plating experiments demonstrated that the bacteria presumably responsible for lactic acid removal could not survive in our system at lower pH. This suggests that the coexistence of yeast and bacteria in the reactor can be explained by the dependence of bacteria on nutrients excreted by the yeast. It is known that *Lactobacilli* only grow in rich media supplemented with yeast extract or amino acids (Rombouts et al., 2020), suggesting that interspecies metabolite transfer may have played an important role in the establishment of the microbial community obtained. Although the existence of these types of eukaryotic‐bacterial cocultures is known from the literature (Frey‐Klett et al., 2011), the coexistence in open reactor systems is relatively unknown and would be an interesting topic for further investigation. It may be suggested that the *Lactobacilli* found a niche in our reactor system by having a slightly higher biomass‐specific substrate uptake rate and by being able to form extracellular polymeric substance biofilm structures within the yeasts' hyphae tangles (Oleksy & Klewicka, 2018; Tiukova et al., 2014; Zeidan et al., 2017). In such a situation, a balance could occur between the prokaryotes and eukaryotes, while they both contribute to granule and product formation.

### 4.3 Applications of the ethanol‐VFA switch

The production of ethanol in an open granular sludge process may be an alternative to conventional industrial ethanol production processes. However, the achieved ethanol yield in this study was only 0.28 (g ethanol)/(g glucose) while in the conventional ethanol production process, much higher ethanol yields (and concentrations) are obtained, for example, 0.45 (g ethanol)/(g glucose) (Siqueira et al., 2008). To which extent the process presented in this study can be optimized to achieve comparably high yields is a potential direction of future research. One option is a further reduction of the operation pH in the bioreactor to make VFA production even more unlikely. However, since the pK_a_ of VFA is around 4.7, we do not anticipate a large improvement. On the other hand, the reduction of pH 4.0 to pH 3.5 indicated already an encouraging reduction of Y_VFA_ from 0.20 ± 0.04 to 0.13 ± 0.02. Additionally, to obtain a very high ethanol yield, lactic acid production should be further minimized, possibly by the same strategy of reducing the operational pH. A promising application of the open culture combined ethanol/lactate/VFA production presented in this study is the industrial production of the precursors for medium chain length fatty acids (MCFA) production from carbohydrate‐rich waste streams (Kleerebezem et al., 2015). Both ethanol and lactate can be used as an electron donor in the chain elongation process (Angenent et al., 2016). While the production of VFA in many waste streams is often trivial, the availability of an economically attractive source of ethanol as an electron donor can be a challenge. The opportunity to convert organic waste streams into the right proportions of ethanol and VFA may be an opportunity for MCFA production from waste streams without the requirement of the addition of an electron donor from another source.

Another process that may benefit from the control of the ethanol/VFA ratio in a fermented substrate stream is the waste‐based PHA production process. In this process, VFA is known to be the preferred substrate source for the production of PHA biopolymers, while the presence of ethanol can lead to a decrease in the product yield. In that case, substrate fermentation at pH 4.5 or higher is likely beneficial.

## 5 CONCLUSION

A remarkable switch from VFA to ethanol production was observed when lowering the pH in an anaerobic granular sludge reactor from pH 4.5 to pH 4.0. The associated microbial community changed from a bacterial community dominated by *Clostridia* species to an ethanol‐producing consortium of eukaryotic yeasts and prokaryotic *Lactobacilli*. The reactor system performed relatively stable with conversion rates of up to 100 gCOD/(L d) due to effective uncoupling of the solid and liquid retention time. Herewith the low‐pH anaerobic granular sludge process enables high‐rate production of the precursors for the MCFA, producing a chain elongation process.

## AUTHOR CONTRIBUTIONS

Bart Joosse and Kasper de Leeuw did the experimental work. Jelmer Tamis and Robbert Kleerebezem supervised the work. All authors contributed to the data interpretation and the writing of the manuscript.

## Data Availability

The data that support the findings of this study are available from the corresponding author upon reasonable request.

## APPENDIX 1

**Table A1:** Overview of the characteristics of the system from cycle data measurements on day 121 compared to the steady‐state values

Parameters	Steady‐state average values	Cycle experiment values	Units
Y_X_	0.13 ± 0.03	0.09	Cmol/(mol glucose)
Y_ethanol_	1.10 ± 0.07	1.10	mol/(mol glucose)
Y_lactate_	0.29 ± 0.03	0.27	mol/(mol glucose)
Y_acetate_	0.44 ± 0.08	0.45	mol/(mol glucose)
Y_CO2_	1.21 ± 0.06	1.15	mol/(mol glucose)
q_s_^max^	0.20 ± 0.03	0.27	Cmol/(CmolX·h)
μ^max^	0.0045 ± 0.0006	0.0041	/h
Carbon balance	94 ± 4	90	% of total C
COD balance	94 ± 4	90	% of total COD

*Note*: Not shown are products that were present in small quantities (e.g., valerate, butyrate, propionate, succinate, and glycerol).

## APPENDIX 2 PLATING OF REACTOR SPECIES

The agar plates were composed of reactor growth medium supplemented with glucose (58 mM, mimicking reactor conditions) and 1% w/v bacteriological grade agar. The plates containing yeast extract were supplemented with 5 g/L yeast extract. The pH of the agar media was set to pH 3.5–4 before pouring. The poured plates were stored at 30°C in an anaerobic jar that was flushed with nitrogen gas for at least 24 h to ensure anaerobic conditions. The first series of plates were inoculated with 100 μl of mixed reactor biomass using the pour‐plate technique (Sanders, 2012). The plates were incubated in an anaerobic jar that was flushed with nitrogen gas and placed in a shaker at 30°C for 7 days. Colony growth was visually checked every day. After the 7 days period, single colonies were picked from the plates and transferred to secondary plates using the streak‐plate technique. The secondary plates were then incubated again in an anaerobic jar in a shaker at 30°C.
